# The Crime of Vehicular Homicide in Italy: Trends in Alcohol and Drug Use in Fatal Road Accidents in Lazio Region from 2018 to 2024

**DOI:** 10.3390/toxics13070607

**Published:** 2025-07-19

**Authors:** Francesca Vernich, Leonardo Romani, Federico Mineo, Giulio Mannocchi, Lucrezia Stefani, Margherita Pallocci, Luigi Tonino Marsella, Michele Treglia, Roberta Tittarelli

**Affiliations:** 1 Laboratory of Forensic Toxicology, Section of Legal Medicine, Social Security and Forensic Toxicology, Department of Biomedicine and Prevention, Faculty of Medicine and Surgery, University of Rome “Tor Vergata”, Via Montpellier 1, 00133 Rome, Italy; francesca.vernich@uniroma2.it (F.V.); leonardo.romani.09@students.uniroma2.eu (L.R.); f.mineo@med.uniroma2.it (F.M.); giulio.mannocchi@uniroma2.it (G.M.); lucrezia.stefani@students.uniroma2.eu (L.S.); marsella@uniroma2.it (L.T.M.); roberta.tittarelli@uniroma2.it (R.T.); 2 PhD School in Medical-Surgical Applied Sciences, University of Rome “Tor Vergata”, Via Montpellier 1, 00133 Rome, Italy; 3 Faculty of Medicine and Surgery, Link Campus University, Via del Casale di S. Pio V 44, 00165 Rome, Italy; margherita.pallocci@gmail.com

**Keywords:** road homicide, ultra performance liquid chromatography (UPLC-MS/MS), head-space gas chromatography (HS-GC-FID), driving under the influence of alcohol and drugs, cocaine, alcohol, cannabinoids

## Abstract

In Italy, the law on road homicide (Law no. 41/2016) introduced specific provisions for drivers who cause severe injuries or death to a person due to the violation of the Highway Code. The use of alcohol or drugs while driving constitutes an aggravating circumstance of the offence and provides for a tightening of penalties. Our study aims to report on the analysis performed on blood samples collected between January 2018 and December 2024 from drivers convicted of road homicide and who tested positive for alcohol and/or drugs. The majority of the involved subjects were males belonging to the 18–30 and 41–50 age groups. Alcohol, cocaine and cannabinoids were the most detected substances and the most frequent polydrug combination was alcohol and cocaine. We also investigated other influencing factors in road traffic accidents as the day of the week and the time of the day in which fatal road traffic accident occurred, and the time elapsed between the road accident and the collection of biological samples. Our data, in line with the international scenario, strongly support that, in addition to the tightening of penalties, raising awareness plays a key role in preventing alcohol- and drug-related traffic accidents by increasing risk perception and encouraging safer driving behaviors.

## 1. Introduction

Road traffic deaths and severe injuries are still a significant global concern for public health, resulting in an estimated 1.19 million deaths in 2021 [1].

According to the World Health Organization (WHO), road traffic accidents (RTAs) are the leading cause of death and injuries for children and young people aged 5–29 years and are the twelfth leading cause of death involving people of all ages [1].

The “Road Safety Annual Report 2023”, including preliminary data of 26 countries for the first semester of 2024, reports that the number of road victims decreased by 2% in 16 countries in the first half of 2024 compared to 2023 [2]. Table 1 shows the number of road accident victims between the years 2018 and 2023.

The highest number of road accident victims was recorded in 2018 with 25,191 fatalities, while a significant decrease occurred in 2020–2021, years of restrictions due to the COVID-19 emergency. In 2023, 20,365 victims were registered in the European Union (EU), a slight decrease compared to 2022 and 2019, when 20,685 and 22,763 victims were observed, respectively [3]. Furthermore, driving under the influence of alcohol (DUIA) and/or drugs (DUID) is a major factor in fatal RTAs worldwide [4,5]. According to the WHO, about 10.0% of the victims of RTAs are caused by alcohol [1].

Many authors agree on the impact of drunk driving, as alcohol can lead to an overconfidence in driving abilities [6] and impairs several driving-related skills, including cognitive functions [7,8,9], reaction time, coordination, judgement and motor functions [9].

Research studies have also examined the effects of drugs on driving performance [10]. Δ9-tetrahydrocannabinol (THC) reduces motor activity, attention and alters coordination [11,12] as well as causing drowsiness and mood changes [11,13]. The use of stimulants as amphetamines (e.g., methamphetamine, methylendioxymethamphetamine) while driving can initially improve cognitive performance [14], increase energy levels, and reduce restlessness [15,16]. On the other hand, amphetamines can cause drowsiness and lethargy [16], impair divided attention and interfere with peripheral visual perception [17].

The situation in Italy reflects the European trend, as shown in Table 2. As reported by the National Institute of Statistics (ISTAT) in 2018, Italy recorded the highest number of road accident victims (*n* = 3334). In the following years, there was a decrease in 2019 (*n* = 3173), 2020 (*n* = 2395) and 2021 (*n* = 2875). In 2023, 3039 people died in road accidents in Italy (−3.8% if compared to 2022) [3]. Moreover, from 2018 to mid-2024, data on impaired driving in Italy show that out of a total of 1,027,380 road accidents with injuries, in 215,341 cases, one of the drivers was intoxicated by alcohol, and in 27,781 cases, one was under the influence of drugs or psychotropic substances [3].

Table 3 describes the total number of road accident victims from 2018 to 2023, related to gender characteristics. Data highlights the highest percentage of victims among men (*n* = 14,577 victims), while *n* = 3398 of victims were women [3].

The most affected age group among females was the over-55 age group, although an increase in the 20–29 age group has been registered in recent years; for males, the 20–29 and 45–49 age groups were the most affected. The group with a +23.6% increase in road fatalities over 2022 was people aged 75 to 79. A slight increase was also observed in the age groups of 5 to 9 years old and 45 to 49 years old (+1.8%) [3].

Moreover, data on impaired driving in Italy show that out of a total of 56,075 road accidents with injuries, in 4787 cases, one of the drivers was intoxicated by alcohol, and in 1813 cases, one was under the influence of drugs or psychotropic substances [3].

In 2023, ISTAT reported that 8.5% of road accidents were caused by drunk driving and 3.2% by driving under the influence of drugs, values slightly decreasing as compared to 2022 when rates of 9.2% and 3.3% were recorded, respectively [3].

In Italy, the law on road homicide (Law no. 41/2016) introduced specific provisions for drivers who cause severe injuries or death to a person due to the violation of the highway code. The use of alcohol or drugs while driving constitutes an aggravating circumstance of the offence and provides for a tightening of penalties [18].

If a driver is suspected of road homicide, the Italian police forces take the subject to the nearest hospital for the collection of biological samples to perform toxicological analysis. This procedure is essential to evaluate the possible influence of alcohol or drugs on driving [11]. According to the Italian law, the legal limit of blood alcohol concentration (BAC) is set at 0.5 g/L for expert drivers, while for newly licensed drivers (under the age of 21 years or holding a license for less than three years) it is 0.0 g/L [18]. For DUID, Italy has a zero-tolerance policy and, in any way, does not allow driving after taking drugs [11].

The biological matrix collected for toxicological analysis is whole blood that assesses whether the driver was driving under a psychophysical alteration due to the use of alcohol and/or psychoactive substances. In emergency departments, following the collection of biological samples, an immunoenzymatic screening test is performed. This test, as extensively reported, has a false negative rate close to zero, while the false positive rate varies depending on the class of substances being analyzed. Non-negative samples are sent to the forensic toxicology laboratories for confirmatory testing, to obtain both qualitative and quantitative information on the detected substances.

According to the Decree of the Commissario ad Acta no. U00288/2016, the Forensic Toxicology Laboratory of the University of Rome “Tor Vergata” has been appointed as one of the reference laboratories for road homicides that occur throughout the Lazio region.

This study aims to report on the analysis performed at our laboratory of forensic toxicology on blood samples collected between January 2018 and December 2024 from drivers convicted of road homicide. We also investigated the prevalence and the trend of drug and alcohol abuse over the years, focusing on age and sex characteristics of the drivers and on several factors influencing road traffic accidents (e.g., the day of the week and the time of the day in which fatal RTAs occurred, and the time elapsed between the road accident and the collection of biological samples).

## 2. Materials and Methods

### 2.1. Sample Collection

A total of *n* = 185 blood samples were analyzed between 1 January 2018 and 31 December 2024 at the Forensic Toxicology Laboratory of the University of Rome “Tor Vergata”. The samples belonging to drivers convicted of drugged or drunk driving resulting in death or severe bodily injuries (with a prognosis greater than 40 days), as established by Law no. 41/2016.

The blood tests were mandatory, and the data obtained were aggregated and pseudo-anonymized (samples are coded at the time of sampling) as they were collected for non-medical purposes.

Our laboratory confirmed the non-negative results of the screening tests performed on drivers admitted to the emergency department (ED) after the RTA. Three samples of whole blood were collected under a strict chain of custody procedure. Each tube was labelled with tamper-evident seals and identified separately with the letters “A”, “B” and “C” to guarantee the integrity and the security of the specimens and to avoid manipulation and conduct or statements that could be viewed as offensive or inappropriate [19]. The sample “A” was analyzed by the ED and if the screening tested non-negative for alcohol or drug abuse, sample “B” was sent to our laboratory for confirmation tests. Sample “C” was kept frozen and available for eventual counter analysis.

### 2.2. UPLC-MS/MS Chemicals and Reagents

#### 2.2.1. Blood Sample Preparation

The detection and quantification of psychotropic substances in whole blood relied on a protein precipitation step for sample preparation. A volume of 100 µL of blood was added with 100 μL M3 line^®^ Comedical reagent (stabilizing agent), 5 μL IS working solution (IDS4 BS—Drugs and Pharmaceuticals—Deuterate Internal Standard mix—Blood and Saliva—Comedical^®^, Comedical S.r.l., Trento, Italy) and 400 μL Precipitating Solution Comedical M3 line^®^ multiMATRIX (precipitating agent).

The samples were centrifuged at 3000 rpm for 5 min, and the supernatants were collected and evaporated to dryness at a temperature of 25 °C under a gentle nitrogen flow. The dried samples were reconstituted with 200 µL of mobile phase 1:1 (aqueous A1 mobile phase Comedical^®^/organic B2 mobile phase Comedical^®^) and 3 µL were injected directly into the chromatographic system.

#### 2.2.2. Calibrators and Quality Control Solutions

Calibrator levels were prepared by spiking with different volumes of a working solution containing 20 non-deuterated analytes in blank whole blood. All standards of the target analytes described in Table 2 were purchased from LGC Standards. The calibrators were on five levels, with ranges from the lower limit of quantification (LLOQ) to 200 ng/mL for amphetamines and phencyclidine, LLOQ to 100 ng/mL for cocaine, cocaine metabolites and opiates, and LLOQ to 20 ng/mL for buprenorphine and cannabinoids, except THC-COOH from LLOQ to 50 ng/mL, respectively (Table 4). The respective LOD (limit of detection) and LLOQ are reported in Table 5.

The IS working solutions were prepared in methanol from the IS stock solutions at 0.1 mg/mL. The IS working solutions had the following concentrations, respectively: 5000 ng/mL for amphetamine-d6, MDA-d5, methamphetamine-d5, MDA-d5, MDMA-d5, MDEA-d5, 500 ng/mL for THC-d3 and 11-OH-THC-d3, 1250 ng/mL for THC-COOH-11-nor-Δ9-THC carboxylic acid-d3, and 2500 ng/mL for cocaine-d3, benzoylecgonine-d3, cocaethylene-d3, codeine-d3, methadone-d3, EDDP-d3, morphine-d3, 6-O-monoacetylmorphine-d3.

Three lyophilised quality controls (QC) were used at three different concentrations (Comedical^®^, Trento, Italy): WB blank, WB 20 positive low and WB 20 positive high, respectively (Table 4). The WB QCs were reconstituted in deionised water and stored at −20 °C.

### 2.3. HS-GC-FID Chemicals and Reagents

#### 2.3.1. Reagents and Sample Preparation

All the reagents used for the analyses were analytical reagent grade. Sodium fluoride (NaF) was purchased by Labochimica s.r.l. (Campodarsego, Padua, 35011, Italy), 2-propanol, used as internal standard (IS), was purchased from PanReac© AppliChem GmbH (Ottoweg 4, D-64291, Darmstadt, Germany). Deionized water was produced from a MINIDIA Plus system (Quality Invents, Arluno, Milan, Italy) with 10 MΩ·cm output.

For sample preparation, 500 μL of whole blood was placed into a 20 mL glass headspace vial and was added with 1% NaF, used as a preservative to prevent ethanol neo-formation and 500 μL of IS (2-propanol 0.5 g/L). The samples were sealed with aluminum crimp seals with PTFE/silicone septa, and then injected into the GC system.

#### 2.3.2. Calibration and Quality Controls

Ethanol aqueous solutions (LGC Standards, Queens Road, Teddington, Middlesex, TW11 0LY, UK) and ethanol controls in whole blood (ACQ Science GmbH, Etzwiesenstrasse 37, D-72108 Rottenburg-Hailfingen, Germany) were used as reference material for calibration and quality controls. Both calibrators and controls were stored at 4 °C.

For each analytical session, calibrator working solutions were prepared in water from the certified reference material (CRM) at eight different concentration levels (0.1 g/L; 0.25 g/L; 0.5 g/L; 0.8 g/L; 1.0 g/L; 1.5 g/L; 2.0 g/L and 3.0 g/L). Low, medium and high-quality controls (QCs) (0.3 g/L; 1.25 g/L; 2.5 g/L) were prepared daily in aqueous solution from the stock solution (4.0 g/L).

### 2.4. UPLC-MS/MS Analysis

Analysis for the detection and quantification of psychotropic substances was performed with a UPLC Acquity H Class (Waters, Milford, MA, USA) coupled to a tandem quadrupole mass spectrometer (XEVO TQD, Waters, Milford, MA, USA). The analytes were separated on a Waters Atlantis™ Premier BEH C18 AX Van Guard™ FIT (2.5 μm 2.1 × 100 mm) (Waters, Milford, MA, USA) column, set at a temperature of 50 °C. The chromatographic run lasted for 11 min and the gradient elution was performed with two mobile phases, A1 (aqueous solution) and B2 (organic solution), at a flow rate of 0.4 mL/min. The initial conditions were 90:10 (A1/B2). Phase A gradually ramped down from 90% to 0% and phase B gradually ramped up from 10 to 100% in 7 min and it was held for 1 min before returning to initial conditions over the next 3 min. Mass spectrometric analysis was performed in positive ion multiple reaction monitoring (ES+ MRM) mode. Two transitions for each analyte and deuterated standards were found (except for Amphetamine-d6, Methadone-d6, 11-OH-THC-d3 and THCCOOH-d3). Transitions, relative cone voltage (V) and collision energy (eV) are reported in Table 6 for all the analytes under investigation.

Data analysis was performed with the Waters TargetLynx XS V. 4.2 SCN1045.

### 2.5. HS-GC-FID Analysis

Analysis for the detection and quantification of ethanol was performed with an Agilent 7820A gas chromatograph-flame ionization detector (GC-FID) equipped with an Agilent DB-ALC-2 capillary column (30 m × 320 µm I.D. × 1.2 µm thickness) and an Agilent 7694E Headspace sampler (Agilent Technologies, Palo Alto, CA, USA). The data analysis was performed with the Agilent OpenLAB CDS ChemStation Edition for GC System (REV.C.01.07 (27)).

The blood samples were incubated at a temperature of 37 °C for 10 min before the injection. The GC injector temperature was set at 200 °C. An isothermal temperature condition at 35 °C in the oven was held throughout the GC run (7 min). The constant flow of H_2_ and air was 40 mL/min and 400 mL/min, respectively. A split ratio of 10:1 mode was used, and split flow was set at 25 mL/min. The detector temperature was 250 °C. A volume of 1 μL was injected into the GC-FID system.

### 2.6. Method Validation

#### 2.6.1. UPLC-MS/MS

The method was fully developed and validated in accordance with updated established international criteria [20,21]. Accuracy, precision, selectivity and carry-over were calculated by injecting five different daily replicates of calibration points and five replicates of quality control (QC) samples. Dilution integrity was tested for over-the-curve samples with a concentration 10 and 50 times higher than the highest calibrators, with a dilution in mobile phase before sample treatment, verifying precision and accuracy to be within 15.0%. Process efficiency (PE), matrix effect (ME) and recovery were obtained according to the experimental design proposed by Matuszewski et al. [22].

The method presented here allowed the detection of all the target analytes with a run of 11 min after a simple treatment of the samples. No additional peaks due to endogenous substances and carryover interfering with analytes and ISs were detected. The method was linear for all analytes under investigation, with a correlation coefficient (r^2^) always better than 0.992. Limit of detection (LODs) ranged from 0.05 ng/mL to 0.36 ng/mL in blood, while LOQs ranged between 0.15 and 1.19 ng/mL in blood.

Process efficiency for all analytes was always better than 68.9% and ion suppression due to matrix effect was within ±25%. Recovery for each substance was always better than 70.1%. Intra-assay and inter-assay precision were always lower than 15% (CV%). The bias never exceeded ±15% (±20% near LLOQ).

#### 2.6.2. HS-GC-FID

The method for quantitative analysis of ethanol in blood was developed and validated in terms of linearity, LLOQ (low limit of quantification), LOD (limit of detection), recovery, precision (CV%) and accuracy (bias) [23]. 

The range of linearity was 0.1–3.0 g/L, with a correlation coefficient (r2) of 0.998.

LLOQ and LOD were 0.1 g/L and 0.03 g/L, respectively, and the recovery was 91%. The intra-assay precision (CV%), inter-assay precision (CV%) were less than 15% and the bias was −7%.

### 2.7. Data Analysis

Data analyses and graph preparation were performed with Microsoft Excel^®^ 2016 MSO (16.0.4738.1000) (Microsoft Corporation^®^, Redmond, WA, USA).

## 3. Results

Our study was performed on blood samples collected at the emergency departments from *n* = 185 drivers alleged in fatal RTAs or severe crash-related bodily injuries while driving under the influence of drugs and/or alcohol. Our laboratory carried out the confirmatory tests on presumptive non-negative samples based on the results of the screening performed at the emergency departments. We reported only non-negative samples collected from the Lazio region, ranked second among all Italian regions in terms of the number of driving licenses. This area was chosen as it represents our reference population and reflects our routine clinical and forensic practice.

**Figure 1:**
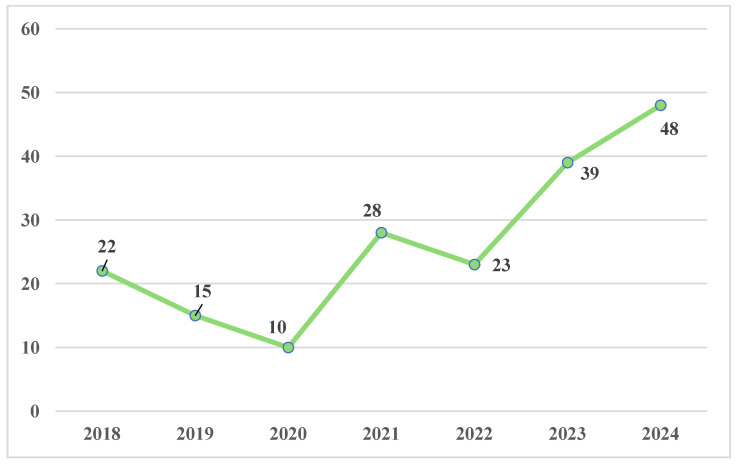
Blood samples analyzed from 2018 to 2024.

Data reported in Figure 1 showed a progressive decrease in the number of cases from 2018 (*n* = 22) to 2020 (*n* = 10), likely related to the COVID-19 emergency. An increase in the number of drugged or drunk drivers was registered in 2021 (*n* = 28), followed by an initially steady trend in 2022 (*n* = 23) that increased in the following years, with *n* = 39 cases in 2023 and peaking in 2024 with *n* = 48 drivers involved in fatal road accidents while impaired by alcohol or drugs.

### 3.1. Epidemiological Data

Most of the drivers were males (*n* = 154), while females accounted for the remaining (*n* = 31).

**Figure 2:**
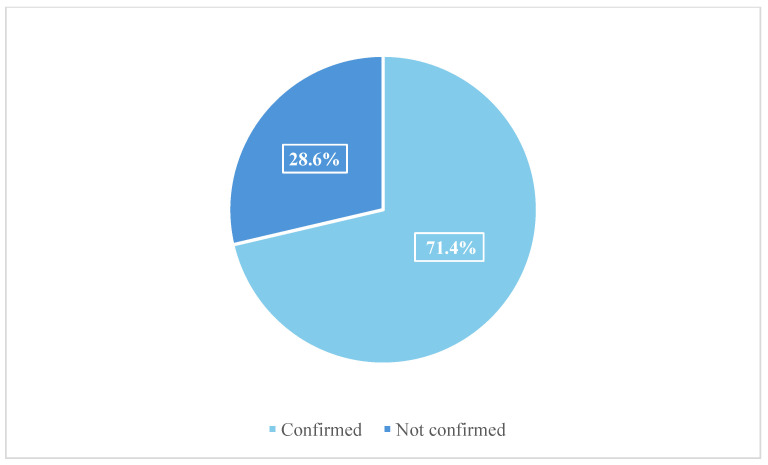
Total number of cases confirmed with UPLC-MS/MS and/or HS-GC-FID analysis.

Out of *n* = 185 cases, *n* = 132 were confirmed for the presence of alcohol and/or drugs, whereas *n* = 53 cases were not confirmed (Figure 2).

Out of the confirmed cases, *n* = 114 were males (86.4%) and *n* = 18 were females (13.6%).

Table 7 shows the data on the average age of males and females who tested positive for toxicological analyses. The average age of males was 35 years (mean 34.8 ± 13.0) with a minimum age of 15 years and a maximum of 76 years; for females, the average age was 37 years (mean 36.7 ± 13.1), with a minimum age of 21 years and a maximum of 74 years.

Data also showed a prevalence of drivers in the 18–30 age group (*n* = 54), followed by 41–50 (*n* = 31) and 31–40 (*n* = 29) age groups.

### 3.2. Toxicological Results

Data were processed, splitting the cases into two groups: group A consisted of *n* = 95 cases (72.0%) that tested positive for one class of substance and group B included *n* = 37 cases (28.0%) of drivers who tested positive for more than one class of substances. Our study was conducted according to the guidelines established by the Commissioner’s Decree No. U00288 of 27 September 2016. This decree outlines detailed procedures to perform toxicological analyses in cases of fatal car accidents and serious personal injuries (articles 589-bis and 590-bis of Law No. 41/2016) and specifies the classes of substances that must be tested in cases of alcohol intoxication or impairment due to psychoactive substances. In these paragraphs, the names of the individual substances (active agents) are used to refer also to their metabolites (e.g., “cocaine” also includes benzoylecgonine).

In group A, ethanol was the most prevalent substance (*n* = 33; 34.7%), followed by cocaine (*n* = 28; 29.5%) and cannabinoids (*n* = 20; 21.1%). The number of cases tested non-negative for opiates was *n* = 12 (12.6%), of which *n* = 11 were positive for morphine and *n* = 1 for codeine. Midazolam and diazepam were each detected in *n* = 1 case. These substances were identified and quantitatively determined using gas chromatography-mass spectrometry (GC-MS). These analyses were carried out in relation to the documented in-hospital administration of the aforementioned drugs, as recorded in the patient’s medical chart. The non-negativity of these two substances was confirmed because medical doctors did not report their administration in hospital settings.

Group B included *n* = 37 cases, in *n* = 33 cases (89.2%) the drivers were tested positive for two classes of substances, in *n* = 3 cases (8.1%) for three classes and in *n* = 1 case for four substances. The most observed association in group B was between alcohol and cocaine (*n* = 12; 32.4%), followed by cocaine and cannabinoids (*n* = 10; 27.0%) and cannabinoids and alcohol (*n* = 4; 10.8%). We also observed several other minor associations between cannabinoids and morphine (*n* = 1; 2.7%), morphine and methadone (*n* = 1; 2.7%), cocaine and morphine (*n* = 2; 5.4%), cocaine and methadone (*n* = 1; 2.7%) and cocaine and midazolam (*n* = 2; 5.4%). In three cases in which the positivity to three substances was confirmed, the detected compounds were alcohol, cocaine and ∆9-THC (*n* = 3; 8.1%). The compounds revealed in the subject tested positive for four substances were alcohol, cocaine, cannabinoids and morphine (*n* = 1; 2.7%).

### 3.3. Factors Influencing Road Traffic Accidents

To identify risk factors for road accidents, several characteristics were studied, including the day of the week and time of day when the accident occurred and the time interval between the accident and the collection of blood for toxicological analyses.

The days were divided into two main groups: weekdays (WD), which included all road accidents that occurred from midnight on Monday to 23:59 on Thursday, and weekend days (WE), which included road accidents that occurred from midnight on Friday to 23:59 on Sunday.

The results showed that *n* = 82 RTAs (44.3%) occurred in WD and *n* = 95 (51.4%) in WE. For *n* = 8 RTAs (4.3%), we did not have sufficient information.

**Figure 3:**
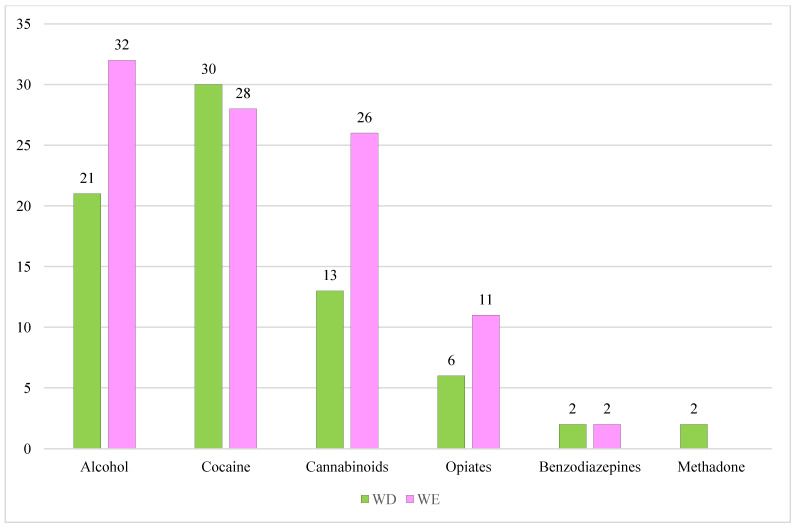
Total number of psychoactive substances detected in fatal RTAs split in WD and WE.

We also studied the distribution of the different classes of substances detected in UPLC-MS/MS confirmation analysis, split by WD and WE (Figure 3).

In the WD group, cocaine was the most detected drug with *n* = 30 cases (40.5%), followed by alcohol with *n* = 21 cases (28.4%), *n* = 13 cases tested positive for cannabinoids (17.6%), *n* = 6 for opiates (8.1%), *n* = 2 for benzodiazepines (2.7%), and *n* = 2 for methadone (2.7%).

In the WE group, in *n* = 32 cases the analyses revealed the presence of alcohol (32.3%), in *n* = 28 cocaine (28.3%), in *n* = 26 cannabinoids (26.3%), in *n* = 11 morphine (11.1%), and in *n* = 2 benzodiazepines (2.0%).

#### 3.3.1. Descriptive Analysis of Weekday (WD) Fatal Crashes

Out of *n* = 57 people involved in traffic accidents in WD, *n* = 51 were males (89.5%) belonging mainly to the age group 41–50 (*n* = 20; 39.2%) followed by the 18–30 age group (*n* = 15; 29.4%), and the 31–40 age group (*n* = 9; 17.7%). *N* = 3 people were over 60 years (5.9%), *n* = 2 were between 51–60 years old (3.9%) and *n* = 1 subject was under 18 years old (2.0%).

**Figure 4:**
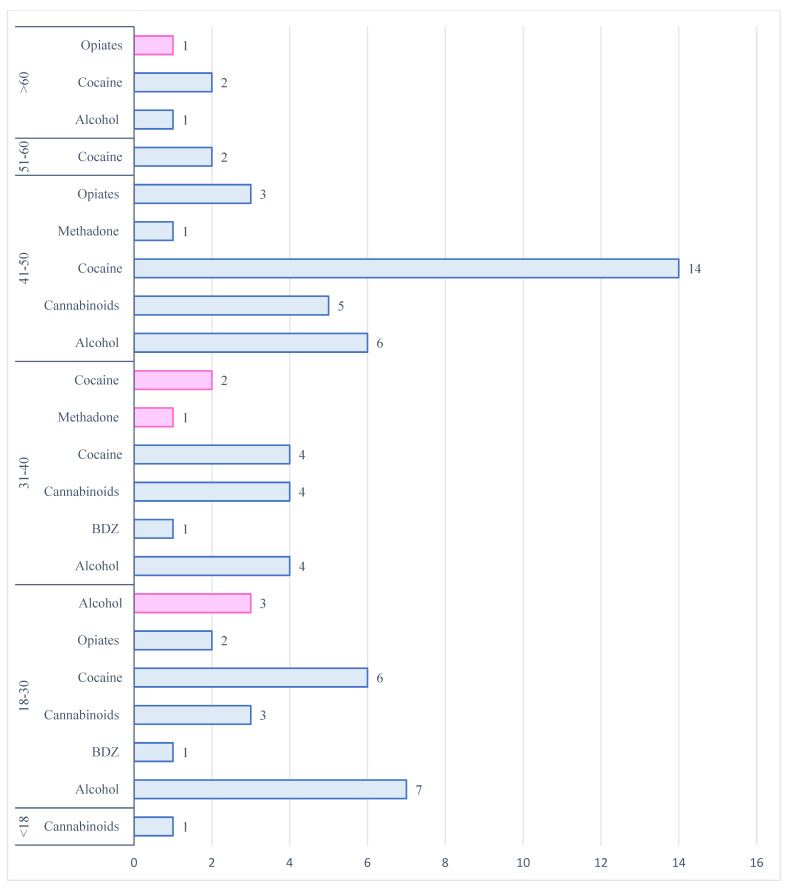
The distribution of psychotropic substances involved in a fatal RTA during WD split by age group and gender (blue for males and pink for females).

The most detected substance in the male group was cocaine (*n* = 28; 41.8%), followed by alcohol (*n* = 18; 26.8%), cannabinoids (*n* = 13; 19.4%), opiates (morphine) (*n* = 5; 7.5%), benzodiazepines (midazolam) (*n* = 2; 3.0%) and methadone (*n* = 1; 1.5%). Figure 4 summarizes the frequency of drugs detected in relation to the age of users.

Only *n* = 6 (10.5%) females were involved in a fatal car accident during WD. In *n* = 3 cases, they belonged to the 18–30 age group (50.0%), in *n* = 2 cases to the 31–40 age group (33.3%) and in *n* = 1 case, the person was over 60 years old (16.7%). In this group, the most widespread substance was alcohol (*n* = 3; 42.9%), followed by cocaine (*n* = 2; 28.6%), opiates (morphine) (*n* = 1; 14.3%) and methadone (*n* = 1; 14.3%).

#### 3.3.2. Descriptive Analysis of Weekend Days (WE) Fatal Crashes

**Figure 5:**
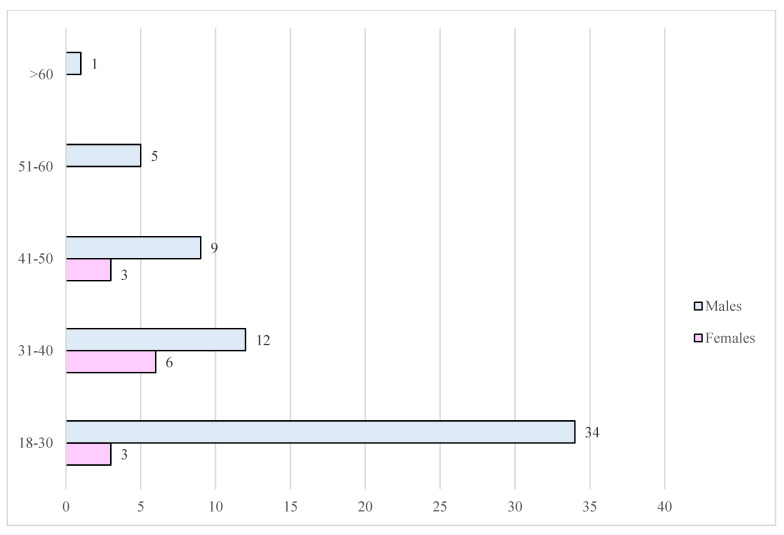
The incidence of fatal RTAs in the WE between the population of drivers split by gender and age.

In the WE group, *n* = 74 were the people involved in a fatal RTA. Out of these, *n* = 62 (83.8%) were males, while *n* = 12 (16.2%) were females (Figure 5).

The age groups most affected were the 18–30 years old (*n* = 34 males; *n* = 3 females; 50.0%), and the 31–40 (*n* = 12 males; *n* = 6 females; 24.3%), followed by the 41–50 (*n* = 9 males; *n* = 3 females; 16.2%), the 51–60 (*n* = 5 males; 6.8%) and the >60 years age groups (*n* = 1 male; 1.4%).

**Figure 6:**
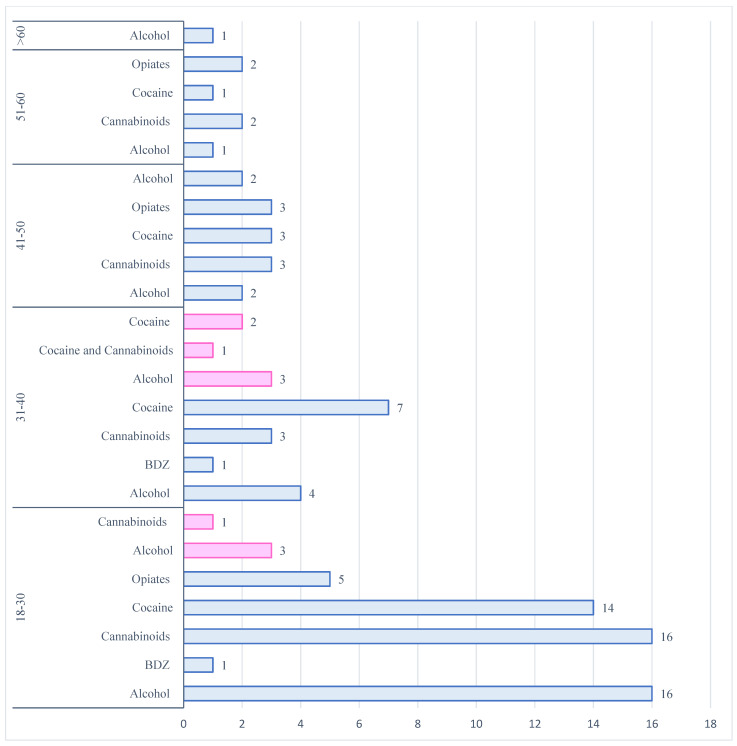
The distribution of psychotropic substances involved in a fatal RTA during WE split by age and gender (blue for males and pink for females).

The most frequently detected substances in the male group were cocaine (29.4%), alcohol (28.2%), and cannabinoids (27.1%) (Figure 6). Opiates were found in 12.9% of cases: the presence of morphine was confirmed in *n* = 10 cases and codeine in *n* = 1 case. Benzodiazepines were identified in *n* = 2 cases (2.4%) (*n* = 1 case midazolam; *n* = 1 case diazepam).

In the female group (*n* = 12), the most frequently abused substance was alcohol (57.2%), followed by cocaine (21.4%) and cannabinoids (21.4%).

The age groups most involved in psychotropic substance abuse were 31–40 years (*n* = 6), 18–30 years (*n* = 3), and the 41–50 group (*n* = 3). These data also include cases of polydrug use.

In the 31–40 age group, *n* = 3 persons tested positive for alcohol; *n* = 2 positive for cocaine and *n* = 1 positive for both cannabinoids and cocaine. *N* = 3 persons tested positive for alcohol in the 18–30 age group and *n* = 1 positive for cannabinoids, and in the 41–50 age group, *n* = 2 cases were positive for alcohol and *n* = 1 positive for cannabinoids.

#### 3.3.3. The Times of Day and the Importance of Timing

**Figure 7:**
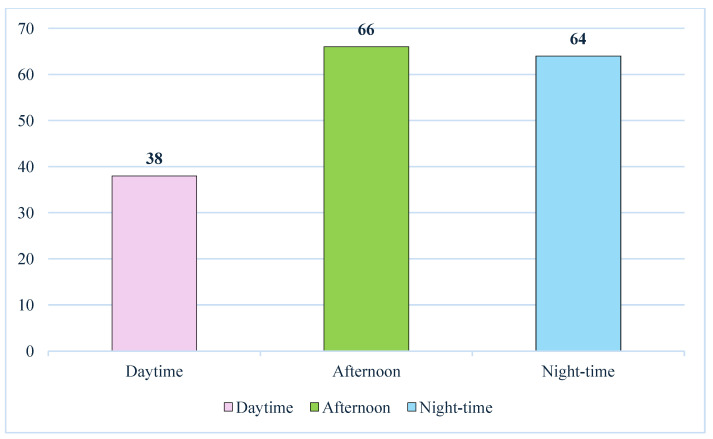
RTAs split in relation to the time of the day.

To better understand the influence of the time in fatal RTAs, the times of day were divided into three intervals: the daytime slot (6:00 a.m.–2:00 p.m.), the afternoon slot (2:00 p.m.–10:00 p.m.) and the night-time slot (10:00 p.m.–6:00 a.m.). A total of *n* = 38 incidents (20.5%) occurred in daytime, *n* = 66 (35.7%) in the afternoon and *n* = 64 (34.6%) in the night-time. For *n* = 17 incidents (9.2%), we did not have sufficient information about the timing of the RTA (Figure 7).

The time interval between RTAs and blood collection was divided into several time groups (0–1 h; 1–2 h; 2–3 h; more than 3 h) to investigate whether the time between the road accident and the sampling could have affected the outcome of the analysis.

Out of the *n* = 132 cases confirmed by UPLC/MS-MS and HS-GC-FID analysis, in *n* = 8 cases (6.0%) the time interval was less than one hour, in *n* = 42 (31.8%) was between one and two hours; in *n* = 41 (31.1%) between two and three hours, and in *n* = 19 cases (14.4%) the time interval was greater than three hours. For *n* = 22 road accidents, (16.7%), we did not have sufficient information.

**Figure 8:**
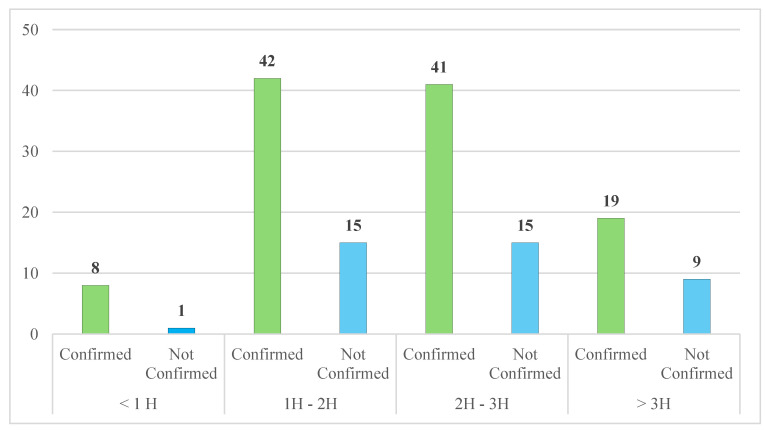
The influence of the time interval in confirmation analysis after fatal RTAs.

Moreover, for the *n* = 53 cases not confirmed by UPLC/MS-MS and HS-GC-FID analysis, in *n* = 15 (28.3%) the interval was between one and two hours and two and three hours; in *n* = 9 (17.0%) was greater than three hours and in *n* = 1 (1.9%) the interval was less than one hour (Figure 8). For *n* = 13 RTAs, (24.5%), we did not have information on the time interval for blood sampling.

## 4. Discussion

The use of psychotropic substances is an ever-increasing public health concern worldwide. Although laws exist worldwide to punish and restrict driving under the influence (DUI) of alcohol and drugs, this issue is still a major cause of death affecting all age groups [1].

Our study aimed to investigate the trend of drug and alcohol use in drivers involved in fatal traffic accidents in the Lazio region, over a seven-year period (2018–2024). 

In such cases, our laboratory exclusively performs confirmatory analyses on individuals who yielded presumptive non-negative results during initial screening. Consequently, data processing was limited to samples that tested positive for alcohol or illicit substances, and no control group was included for this reason.

Data showed that fatal RTAs have increased since 2020 (*n* = 10), with a peak in 2024 (*n* = 48). More deeply, comparing our results with European and ISTAT data, we highlighted a comparable trend between the years 2018 and 2021. In 2022, we registered a slight decrease in the number of road accident cases compared with published data. In 2023, we described the opposite trend recording a modest increase, while on the other hand, a decreasing tendency was observed.

Out of *n* = 185 cases, males were mostly involved in fatal RTAs (*n* = 154) than females (*n* = 31). The mean age of the people implicated in fatal RTAs was 36 years.

The most vulnerable age group was 18–30 years (*n* = 54), followed by 41–50 years (*n* = 31) and 31–40 years (*n* = 29). Our data are in agreement with the “World report on road traffic injury prevention” and other studies, which stated that younger age groups driving cars have a higher risk of being involved in fatal or non-fatal road accidents than older people [24,25,26].

Alcohol (*n* = 33), cocaine (*n* = 28) and cannabinoids (*n* = 20) were the most detected substances in the study population that tested positive for a single class of substances.

The most frequent polydrug use was represented by the combination of alcohol and cocaine (*n* = 12), cocaine and cannabinoids (*n* = 10) and alcohol and cannabinoids (*n* = 4). These findings are consistent with those reported by the European Union Drugs Agency (EUDA) which identifies cannabis and cocaine as the most commonly used illicit substances in Europe [27].

The use of alcohol and drugs can also be related to the days of the week [5], with an increased risk of RTAs with fatal consequences during WE and in the afternoon/night-time periods. One of the increased risks of RTA in WE is binge drinking. This refers to heavy episodic drinking of five or more drinks per occasion for men or four or more drinks for women [28,29,30]. Binge drinking is particularly prevalent during the evening and night-time hours, especially among young people who have an increased risk of fatal traffic accidents due in part to their less driving experience.

Our data showed that the highest number of RTAs occurred in the WE (*n* = 95), and especially involved males (*n* = 62) in the 18–30 age group (*n* = 34), with a prevalence of alcohol and cannabinoids use (*n* = 16) followed by cocaine (*n* = 14). Conversely, the most affected age group for females (*n* = 12) involved in fatal RTAs in WE was 31–40 years, with *n* = 6 cases, with a prevalence of use of alcohol and cocaine (*n* = 3 and *n* = 2, respectively). In *n* = 1 case, a combined use of cannabinoids and cocaine (*n* = 1) was observed.

Our study also considered the time interval between the fatal accident and the collection of biological samples: only in *n* = 8 cases the time distance was less than an hour, while in most cases the collection occurred between 1–2 h, or after 3 h. These data highlight the importance of the timely collection of biological samples from the drivers and the exigency of maximum collaboration between law enforcement and health care providers to prevent drug or alcohol concentrations from falling below legal limits or active ingredients from being metabolized into their inactive metabolites that have no biological effects. This means that while the presence of the metabolites can indicate drug use, they do not provide information about the effects of the drug on the body at the time of the accident or the person’s altered state. We highlight the critical issue of the delay between the accident and the collection of blood samples. The reform of Articles 186 and 187 of the Italian Highway Code, following the amendments introduced by Law No. 177 of 2024, introduces substantial changes aimed at strengthening road safety. The ultimate goal is to raise awareness among law enforcement officers and medical and paramedical personnel working at the scene of the accident, with the aim of drastically reducing operational delays in taking samples and facilitating faster access to emergency departments.

### Limitations

This study has several limitations that should be taken into account when comparing it to other investigations. This paper was based on the analysis of *n* = 185 blood samples from the Lazio region of Italy. Nevertheless, we concluded that the geographic limitation and the limitate number of cases may affect the ability to generalize the results to other Italian regions or countries. The study was conducted by analyzing the psychoactive substances consistent with current Italian law. Furthermore, the time elapsed between the accident and blood sample collection significantly influences the concentration of the substance in blood, which can complicate the reliable assessment of alcohol or drug-related psychomotor impairment at the time of the accident. Finally, the study focused only on drivers who tested positive for alcohol or drugs, not on drivers in similar accidents who were not under the influence of psychoactive substances.

## 5. Conclusions

Worldwide, the complexity of the phenomenon of driving under the influence (DUI) of alcohol and drugs has become increasingly relevant in recent years. A wide oversight, including countries all over the world, represents the starting point for a complete comprehension of the DUI setting.

Australia has a zero tolerance policy for drug driving [31], as well as Canada, especially for young drivers. The United States also has zero tolerance laws [31], although since 2023, following the introduction of the legalization of recreational use of cannabis for people over 21 years of age [32,33], an increase in road accidents with serious and fatal injuries was recorded and cannabis was detected in 25.1% of the biological samples of the drivers [33,34].

In Europe, Norway adopted a “Vision Zero” strategy in 2001 with severe restrictions for road safety as the reduction in blood alcohol concentration (BAC) from 0.05% to 0.02%, and the introduction of legal limits for narcotics [35,36].

Even in Italy, there is a zero tolerance for drugged and drunk driving, and the Italian government has recently updated the issue of road safety with the law of 25 November 2024, no. 177.

This law further tightens the penalties for people driving while impaired by alcohol, with an increase in the time of license suspension. For repeat offenders, the law mandates the installation of an “alcolock” device in the car, to prevent the car from starting if the driver’s breath alcohol content (BrAC) exceeds the legal limit [37]. Zero tolerance is also enforced for drugged drivers: in this case, it will no longer be necessary to ascertain the state of psychophysical impairment, but a positive result on an oral fluid test will be enough to establish drug use and trigger the suspension of the driving license [37].

Despite traffic laws, it is also important to emphasize the necessity to improve the panel of narcotic and/or psychotropic substances investigated by forensic toxicology laboratories and hospitals, because the drug market is constantly evolving. The European Union Drugs Agency (EUDA) reported that at the end of 2023, over 950 new psychoactive substances were monitored in Europe, 26 of which were first described. Moreover, seven new synthetic opioids and four semi-synthetic cannabinoids have been reported to the EU Early Warning System in 2023 [27].

The EUDA also reported that since 2022, the number of polydrug consumptions was steadily increasing and that most of the individuals who accessed emergency departments for acute intoxication had used cocaine in combination with ketamine [27].

This finding is also supported by J. Palamar’s study, which reported an upward trend in the use of “Tusi” in Latin America and Europe. Tusi”, a phonetic translation of “2C,” a series of psychedelic phenethylamines, is also known and sold as “pink cocaine”, a mix of drugs that contains ketamine and MDMA or in addition methamphetamine, cocaine, opioids and/or new psychoactive substances [38,39] causing serious adverse health effects in the users.

There is a significant need to increase educational efforts targeting youth and making them aware of the correct behaviors to adopt on the roads and the dangers of driving under the influence of alcohol and/or drugs. This is crucial to avoid road accidents that can cause serious health problems both for the drivers and others involved.

## Figures and Tables

**Table 1:** Total number of road fatalities in Europe (2018–2023).

Year	Number of Road Accident Victims
2018	25,191
2019	22,763
2020	18,861
2021	19,948
2022	20,685
2023	20,365

**Table 2:** Total road fatalities in Italy (2018–June 2024).

Year	Total of Road Deaths	Total of Road Accidents	Art. 186 Traffic Laws (DUIA)	Art. 187 Traffic Laws (DUID)
2018	3334	172,553	39,208	5404
2019	3173	172,183	42,485	5340
2020	2395	118,298	25,902	3831
2021	2875	151,875	31,022	4289
2022	3159	165,889	37,678	4608
2023	3039	166,525	39,046	4309
2024 (January–June)	1429	80,057	n.a.	n.a.
Total	19,404	1,027,380	215,341	27,781

Legend: n.a.: not available.

**Table 3:** Total road fatalities in Italy (2018–June 2024) related to gender characteristics.

Year	Total of Road Deaths	Males	Females
2018	3334	2673	661
2019	3173	2566	607
2020	2395	1947	448
2021	2875	2396	479
2022	3159	2579	580
2023	3039	2416	623
2024 (January–June)	1429	n.a	n.a
Total	19,409	14,577	3398

Legend: n.a.: not available.

**Table 4:** Calibration points and quality controls of analytes collected by class.

Calibration Points	Level 1 (ng/mL)	Level 2 (ng/mL)	Level 3 (ng/mL)	Level 4 (ng/mL)	Level 5 (ng/mL)	Quality Controls	WB 20 LOW (ng/mL)	WB 20 HIGH (ng/mL)
Amphetamines	LOQ	10	20	50	200	Amphetamines	30	120
THC and 11-OH-THC	LOQ	1	2	5	20	THC and 11-OH-THC	3	12
THCCOOH	0.62	2.5	5	12.5	50	THCCOOH	7.5	30
Cocaine and metabolites, methadone, EDDP and opiates	LOQ	5	10	25	100	Cocaine and metabolites, methadone, EDDP and opiates	15	60

**Table 5:** LOD and LLOQ of analytes collected by class.

Analytes	LOD (ng/mL)	LLOQ (ng/mL)
Amphetamines	0.18	0.60
THC and metabolites	0.20	0.70
Cocaine and metabolites	0.19	0.70
Methadone	0.20	0.70
EDDP	0.36	1.19
Morphine	0.05	0.15
Codeine and 6-MAM	0.23	0.75

**Table 6:** Retention times, cone voltage, collision energy and MRM transitions of the analytes under investigation.

Analyte	Retention Time (min)	CV (V)	Quantifier MRM Transitions (*m*/*z*)	CE (eV)	Qualifier MRM Transition (*m*/*z*)	CE (eV)
Morphine-d3	0.60	35.00	289.2 > 61	28.00	289.2 > 201	40.00
Morphine	0.60	35.00	286 > 165.1	40.00	286 > 153	40.00
Codeine-d3	0.88	30.00	303 > 215.1	25.00	303 > 61.1	27.00
Codeine	0.88	30.00	300.1 > 215.1	25.00	300.1 > 199.2	27.00
Amphetamine-d6	1.14	20.00	150.1 > 91.1	12.00	–	–
Amphetamine	1.14	20.00	136.1 > 119.1	8.00	136.1 > 91.1	15.00
Methamphetamine-d5	1.20	20.00	154.8 > 91.8	12.00	154.8 > 121.1	10.00
Methamphetamine	1.20	20.00	150.1 > 91.1	12.00	150.1 > 119.1	10.00
MDA-d5	1.23	20.00	185.1 > 110	26.00	185.1 > 163.1	20.00
MDA	1.23	20.00	180.1 > 133.1	18.00	180.1 > 163.1	10.00
6-MAM-d3	1.25	30.00	331 > 61.1	30.00	331 > 195.1	36.00
6-MAM	1.25	30.00	328.1 > 165.1	40.00	328.1 > 181.2	40.00
MDMA-d5	1.28	20.00	199.1 > 165.1	12.00	199.1 > 135.2	20.00
MDMA	1.28	20.00	194.1 > 163	12.00	199.1 > 135.2	20.00
MDEA-d5	1.51	20.00	213.1 > 163.1	14.00	213.1 > 105.1	26.00
MDEA	1.51	20.00	208.1 > 163.2	14.00	208.1 > 135.1	14.00
Cocaine-d3	2.06	30.00	307 > 184.7	20.00	307 > 84.8	30.00
Cocaine	2.06	30.00	304.2 > 182.2	20.00	304.2 > 82.3	28.00
Cocaethylene-d3	2.59	30.00	321.1 > 199.1	20.00	321.1 > 85	30.00
Cocaethylene	2.59	30.00	318.1 > 196.1	20.00	318.1 > 82.1	30.00
Benzoylecgonine-d3	3.10	30.00	293.1 > 171.1	20.00	293.1 > 105.1	32.00
Benzoylecgonine	3.11	30.00	290.1 > 168.1	20.00	290.1 > 105.1	33.00
EDDP-d3	3.31	30.00	281.3 > 235.1	30.00	281.3 > 250.2	22.00
EDDP	3.31	45.00	278.2 > 234.2	26.00	278.2 > 186.2	35.00
Methadone-d3	4.11	30.00	313.3 > 268.2	14.00	–	–
Methadone	4.11	30.00	310.3 > 265.2	14.00	310.3 > 105.1	32.00
11-OH-THC-d3	6.39	30.00	334.2 > 196.1	30.00	–	–
11-OH-THC	6.41	30.00	331.2 > 193.15	24.00	331.2 > 313.1	20.00
THCCOOH-d3	6.82	40.00	348.0 > 195.8	26.00	–	–
THCCOOH	6.84	40.00	345.1 > 193.15	24.00	345.1 > 299.1	26.00
Delta-9-THC-d3	7.35	35.00	318.2 > 123	34.00	318.2 > 196.1	22.00
Delta-9-THC	7.35	35.00	315.21 > 123	34.00	315.21 > 193.1	22.00

**Table 7:** Summary of the minimum, maximum and average age of drivers involved in fatal RTAs between 2018 and 2024 and who tested positive for alcohol and/or drugs.

	Males	Females
Total Cases (*n*; %)	114 (86.4%)	18 (13.6%)
Mean Age	35 years	37 years
Minimum	15 years	21 years
Maximum	76 years	74 years

## Data Availability

The data presented in this study were obtained from the included studies and are openly available.
